# Comparison of carcass condemnation causes in two broiler hybrids differing in growth rates

**DOI:** 10.1038/s41598-023-31422-0

**Published:** 2023-03-14

**Authors:** Merete Forseth, Randi Oppermann Moe, Käthe Kittelsen, Eystein Skjerve, Ingrid Toftaker

**Affiliations:** 1 Norsk Kylling AS, Havneveien 43, 7300 Orkanger, Norway; 2 Norwegian University of Life Sciences, Universitetstunet 3, 1433 Ås, Norway; 3 Animalia, Norwegian Meat and Poultry Research Centre, Lørenveien 38, 0513 Oslo, Norway

**Keywords:** Zoology, Agricultural genetics, Animal breeding

## Abstract

Experimental studies concluded that genetic factors enabling fast growth rate might negatively affect broiler health and welfare. Recently, the proportion of slower-growing broilers has been increasing. However, studies of health in broilers with different growth rates in commercial systems are still scarce. This repeated cross-sectional study aimed to describe causes of carcass condemnations in two broiler hybrids with different growth rates, Ross 308, and Hubbard JA787, and to estimate the effect of hybrid. The study sample consisted of 63,209,415 broilers slaughtered in 4295 batches from 139 farms. All broilers were slaughtered from January 1st, 2015, to June 22nd, 2021, by the same company (Norsk Kylling). All causes of condemnation, except fractures, were more prevalent in Ross 308. The five most common causes (ascites, discolouration, hepatitis, small and skin lesions) were investigated in greater detail, and the effect of hybrid was assessed using mixed effects negative binomial models with the condemnation codes as outcome variables. For the five selected causes, variation in prevalence between slaughter batches was considerable for Ross and minor for Hubbard. The notable differences between hybrids in prevalence and causes of condemnation have important implications for animal health, welfare, economy, and sustainability in broiler production.

## Introduction

In the last 40–50 years, poultry meat has been of increasing importance as a major source of animal protein for the human population^1^. Selective breeding, nutritional improvements and managemental changes have resulted in an efficient broiler chicken that reaches slaughter weight in one third of the time, with higher breast meat yield while consuming only a third the amount of feed compared to hybrids used in the 1950’s^2,3^. The majority of these changes are considered to be related to targeted genetic selection^2^. However, several studies concluded that genetic factors enabling a fast growth rate might negatively affect broiler health and behaviour and, thus, welfare^4^. For instance, studies performed under experimental conditions have reported that fast growth in broilers is a risk factor for high mortality^2,5^, poor adaptive immune system function^6^, increased prevalence of metabolic disorders^7^ and skeletal disorders leading to gait abnormalities and lameness^8^, lower activity levels^5,9,10^, higher scores for foot pad lesions and hock burns^5,10^ and poor feather condition^10^.

Consumer concern for animal welfare has resulted in the development of a market segment using broilers with slower growth rates^11^. Indeed, an increasing proportion of slower growing broilers is used in European broiler production; e.g. 40% in the Netherlands^12^, 11% in the UK^12^ and 23% in France^13^. In Norway, the proportion of slower growing broilers increased to 18% after a complete transition made by a Norwegian broiler company, Norsk Kylling (NK), from the conventional fast growing broiler hybrid Ross 308 to the slower growing hybrid Hubbard JA787 during 2017–2018^14^.

Two studies from year 2020 and 2021 compared broiler chicken hybrids of different growth rates in commercial production systems^15,16^. They found that the slower growing broilers had lower mortality, fewer post-mortem inspection rejections and overall lower gait scores, hock burns and contact dermatitis scores compared to faster growing hybrids^15,16^.These studies also reported more play-, exploration-, comfort- and safety behaviours in the slower growing hybrids^15,16^ indicating beneficial effects of slower growth rates on several aspects relevant to health and animal welfare. However, relevant studies under commercial conditions are still scarce in the scientific literature.

In commercial broiler production, animal health and welfare is commonly evaluated on the basis of registrations made both at the farm (lameness, mortality, culls and behavioural observations) and at the abattoir (plumage cleanliness, condemnations rates and contact dermatitis)^17^. At slaughter, broiler carcasses are subject to meat inspection, and carcasses showing signs of disease or injury rendering the meat unfit for human consumption are condemned. Several common causes of condemnations are disorders that develop over time, such as ascites^4^ and contact dermatitis^4,18^, and a high occurrence might reflect compromised welfare for longer time periods. Thus, slaughterhouse records on causes, and frequency of condemnations are important sources of information regarding the health and welfare status of the broilers throughout the production period. Nevertheless, there is only one study that, to the authors knowledge, has compared carcass condemnation causes between broiler hybrids with different growth rate in commercial broiler production^15^.

To increase the knowledge of health in hybrids differing in growth rate under commercial production conditions, the aim of this study was to describe causes and frequency of carcass condemnation in the two hybrids, Ross 308, and Hubbard JA787, and to estimate the effect of hybrid on the most common causes of condemnation by utilising slaughterhouse records. A subsidiary aim was to assess the effect of slaughter age on carcass condemnation causes in each of the two hybrids.

## Materials and methods

### Study population

The source population of this repeated cross-sectional study consisted of farms contracted to the broiler company Norsk Kylling AS (NK). The study unit was slaughter batch, i.e., all broilers from the same farm and house, slaughtered at the same time. The inclusion criteria were batches consisting of broilers of the hybrids Ross 308 (Ross) and Hubbard JA787 (Hubbard), with time of slaughter in the period from January 2015 to June 2021. All eligible farms were located in two counties in mid-Norway: Trøndelag and Innlandet (a map is included in the supplementary material Fig. S1 online). Owner consent was achieved through digital forms. Non-responders were contacted through either email, SMS or by phone, followed by consent on SMS. The transition to a slower growing hybrid was made over a period of 15 months, starting in July 2017, and completed in October 2018. Consequently, both hybrids were represented during this period. Before July 2017 all production units housed Ross only, and after October 2018 all holdings housed Hubbard.

### Data sources and data management

All broilers included in this study were slaughtered in one slaughterhouse. Records from the broiler company’s database “Production control” were retrieved, comprising flock information and abattoir recordings of all batches slaughtered from January 1st, 2015, to June 22nd, 2021. This database contained information from several sources: Upon arrival at the abattoir, the number of broilers removed before slaughter due to sickness, lesions or dead on arrival were recorded by personnel in the arrival area (to be reported elsewhere). At the slaughter line, post-mortem inspections were made by trained meat inspectors who either approved or condemned the carcass. In case of condemnation, they also recorded the cause of the condemnation. The number of broilers approved for human consumption were registered automatically by an in-line counter where the carcasses are also automatically weighed. The number of animals delivered to slaughter are calculated as the sum of broilers removed before slaughter in the arrival area, slaughtered but condemned broilers and broilers approved for human consumption.

**Figure 1:**
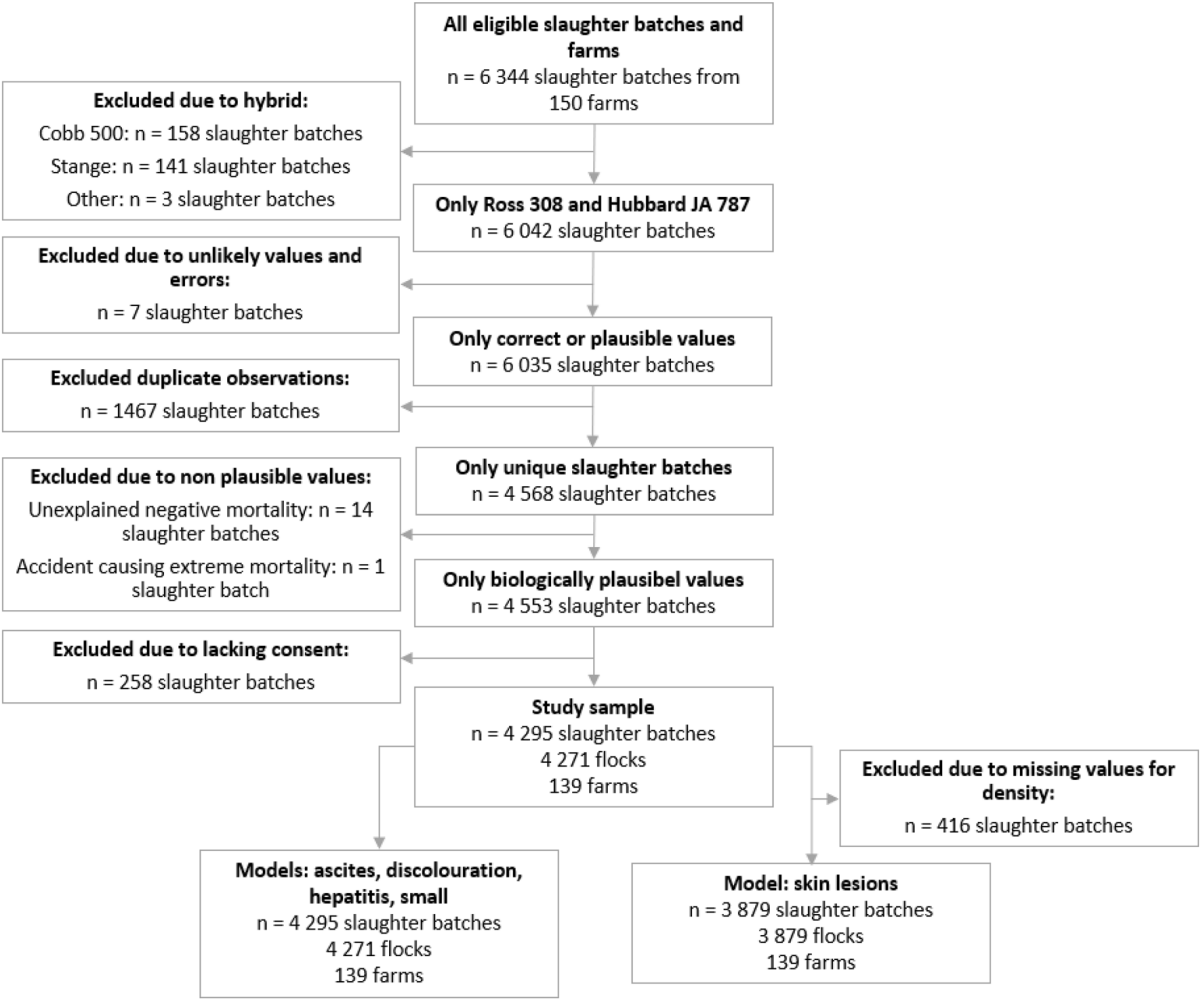
Flowchart describing all eligible slaughter-batches, exclusions, and the final study sample.

The data were stored in Microsoft Excel 365 and exported to Stata SE 17.0^18^ for data management and statistical analysis. Obvious errors, unlikely and missing values were checked against food chain information collected from the farmer or condemnation certificates from the Norwegian Food Safety Authority. Incorrect registrations were replaced with the correct values from these sources. For cases where correct information could not be obtained, the slaughter batch was excluded from the study. A description of all eligible, excluded, and analysed slaughter batches is shown in Fig. 1.

All condemnation codes at the abattoir were recorded as counts per slaughter batch, where a slaughter batch denotes all broilers from the same house and farm slaughtered at the same time. The farms in the present study had one to four houses, and the term flock will be used to address broilers housed together on the same farm during the same time. The hierarchical structure of the dataset along with all retrieved variables are described in Table 1. Only one condemnation code (lesion) could be recorded per bird. For birds with two or more lesions/diseases present, the meat inspector therefore decided which of the lesions were considered most severe and recorded a single code accordingly. The condemnation codes are described in Table 1. To account for seasonal variations, a categorical variable was made based on date of slaughter in the following way: winter: December–February, spring: March–May, summer: June–August and autumn: September–November. Stocking density was measured in kg/m^2^ just before slaughter. A categorical variable for density was made using cut-points at 32.26 kg/m^2^ and 34.39 kg/m^2^ to retrieve groups of approximately equal size, resulting in three categories: low, medium, and high. According to Norwegian legislation the maximum stocking density in a broiler house is 36 kg/m^2^. To avoid too high density at the end of a cycle, part of the flock might be slaughtered followed by the remaining part a few days later, often referred to as thinning.

**Table 1:** Flock information and variable description of the final dataset consisting of 4295 slaughter batches from 139 Norwegian poultry farms in the time period 2015–2021.

Variable	Type of variable	Level in hierarchy	Comments/judgement
Farm ID	Nominal	Level 3 (highest)	Unique identifier of each of the 139 farms
House	Nominal		Every farm had 1–4 houses (median: 1)
Flock ID	Nominal	Level 2	Unique identifier of each flock. Broilers housed together at the same farm. Thinned flocks are registered with one Flock ID
Slaughter ID	Nominal	Level 1 (lowest)	Unique identifier of each batch slaughtered, i.e., all broilers from the same farm and flock, slaughtered on the same day
Hybrid	Dichotomous		Ross 308 or Hubbard JA787
Slaughter date	Continuous		Date for delivery to abattoir
Season	Nominal		Variable for season based on date of slaughter. 1 = winter (December-February), 2 = spring (March–May), 3 = summer (June–August), 4 = autumn (September–November)
No slaughtered	Discrete		The number of broilers delivered to the abattoir
No approved	Discrete		The number of broilers approved for human consumption after ante- and post-mortem control
Density	Ordinal		Stocking density measured in kg/m^2^. Divided into 3 groups, low, medium, and high density for each hybrid. Cut points at 32.26 kg/m^2^ and 34.39 kg/m^2^
Age	Continuous		Age of broilers at slaughter in days. Calculated by subtracting the date of placement on farm from the slaughter date
Ascites	Discrete		Count per batch. Accumulation of fluid in peritoneal cavities or potential spaces. Results in total condemnation
Fractures	Discrete		Count per batch. Old fractures, mainly of wings, accompanied by infection/inflammation. Results in total condemnation
Skin lesions	Discrete		Count per batch. Cellulitis, scratches, or wounds that are deep or infected/inflamed. Results in total condemnation
Hepatitis	Discrete		Count per batch. Visible pathology on the liver. Results in total condemnation
Discolouration	Discrete		Count per batch. Deviant colour or smell that cannot be defined by any of the other condemnation codes. Also includes carcasses with quality defects like wooden breast. Results in total condemnation
Small	Discrete		Counts per batch. Broilers that are considerably smaller than their conspecifics. Results in total condemnation
Total rejects	Discrete		Sum of all carcasses condemned. Total condemnation

### Descriptive statistics

The overall prevalence of each condemnation code was calculated by dividing the total number of condemnations for each code by the total number slaughtered broilers for each of the two hybrids. The within-batch prevalence was calculated by dividing the number of condemnations for each code in a slaughter batch by the number of broilers delivered to slaughter for that batch and is presented by the median and interquartile range for each condemnation code.

### Statistical analysis

#### The effect of hybrid on causes of condemnation

All condemnation causes were recorded as counts per slaughter batch, hence making slaughter batch the unit of the analysis. To assess the effect of hybrid on causes of condemnation, the five most common causes of condemnation were selected as outcome variables and used in further analysis: ascites, discolouration, hepatitis, skin lesions and small (Table 1). In addition, fractures were included in the descriptive assessment of differences between hybrids, as it was the only condemnation cause more prevalent in Hubbard (Fig. 1). The effect of hybrid was assessed using mixed effects negative binomial models for each of the five outcome variables separately. Negative binomial models were chosen over Poisson models due to overdispersion (conditional variance of the outcome variable larger than the conditional mean). The number of broilers submitted to slaughter were set as the exposure variable in all models. To account for the hierarchical structure of the data, farm and flock were included as random effects.

Prior to analysis, the potential causal pathways and links between the exposures and outcome were described and illustrated using directed acyclic graphs (DAGs) (see Supplementary Fig. S2 online). The DAGs were based on narrative literature reviews and biological reasoning, and created using the software Daggity^19^. Variables that were dependent on farm of origin, for example ventilation and nutrition, were considered “farm effects” and were not included in the DAGs. The building of the regression models was based on these causal diagrams. The exposure variable of primary interest was the hybrid, and inclusion of confounders was based on the directed acyclic graphs. Additionally, their importance was assessed by monitoring the change in the coefficient for hybrid when the confounder was included and removed from the model.

For all models biologically plausible interaction effects (age, season, and density) were tested by adding the interaction between each term and hybrid to the model. For the sake of model parsimony, interaction terms were only kept in the model if *p* < 0.01. Regression diagnostics were performed for each model by computing conditional Pearson residuals to determine whether the final models adequately represented the data. Residuals were assessed using normality plots and plotted against predicted values to evaluate the assumption of homoscedasticity. No major shortcomings of the models were detected. To assess the proportion of the unexplained variation in the outcome at the different levels of the hierarchy, we calculated variance proportion components following the method described by Leckie et al^20^.

Exponentiation of the coefficients from negative binomial models are commonly denoted as incidence risk ratios; however, we have in the following chosen to use the term prevalence ratios to adequately describe the effect measure given the cross-sectional nature of the data.

#### The effect of age on causes of condemnation

For Hubbard, the range in slaughter age was 39–50 days, and for Ross the range was 28–37 days. As there was no overlap in the age at slaughter between the hybrids, the effect of age on causes of condemnation was analysed for each hybrid separately. For both hybrids, age was categorised, using a cut point at 45 and 46 days for Hubbard and at 31 and 33 days for Ross, to retrieve groups of approximately equal size. This categorical variable was the main explanatory variable of interest in these models. The effect of age was assessed using mixed effects negative binomial models for each hybrid separately following the same model building approach as described for the analysis of the effect of hybrid. Slaughter batch was the unit of analysis and the outcome variables were: ascites, discolouration, hepatitis, skin lesions and small (Table 1). Farm and flock were included as random effects, and interaction effects for season and density were tested in the model. Model diagnostics were performed in the same manner as described above, and no major shortcomings of the model were detected.

#### Sensitivity analysis

As both hybrids were not present in the dataset throughout the study period, a subset of the data truncated to the time period where there was overlap between the two hybrids (2017–2018) was used in a sensitivity analysis. All models were re-run using this dataset, to evaluate the robustness of the obtained estimates. For the skin lesion model, 3 observations with extreme values (> 300 condemnations) were excluded in this analysis to retrieve model convergence.

## Results

### Descriptive statistics

After cleaning, the final study sample consisted of a total number of 63,209,415 broilers, 34,404,525 Hubbard and 28,804,890 Ross, delivered to slaughter throughout the five-year study period. Only 24/4271 (0.56%) of flocks were thinned. The rest, 4247/4271 (99.4%) flocks, were slaughtered at the same time, i.e., one slaughter batch per flock. This resulted in 4295 slaughter batches from 4271 flocks and 139 farms. Per hybrid, the number of slaughter batches in the study sample were 2428 for Hubbard and 1843 batches for Ross. In total, 62,287,705 carcasses were approved for human consumption, and 921,710 were condemned during the post-mortem control at the slaughterhouse (Table 2).

**Table 2:** Descriptive statistics of 4295 slaughter batches slaughtered during the study period (January 2015–June 2021) for Hubbard JA787 and Ross 308.

	Total no	Slaughter batch				
		Median	Min	Max	Q1	Q3
Hybrid	Hubbard					
No slaughtered	34,404,525	13,677	1675	37,141	11,842	15,203
No approved	34,163,740	13,582	1667	36,781	11,779	15,104
Weight (g)		1654	1279	1968	1589	1707
Age (days)		46	39	50	46	47
Hybrid	Ross					
No slaughtered	28,804,890	15,613	1448	29,841	13,452	17,406
No approved	28,123,965	15,217	1427	29,496	13,090	17,053
Weight (g)		1357	775	1737	1267	1451
Age (days)		33	28	37	32	34

All numbers except total numbers are counts per batch.

The overall median stocking density for all flocks was 33.47 kg/m^2^. For Ross the density was 33.8 kg/m^2^ (2015), 32.0 kg/m^2^ (2016), 33.4 kg/m^2^ (2017) and 32.4 kg/m^2^ (2018). The stocking density for Hubbard changed during the transition period, starting with 32.7 kg/m^2^ in 2017, 33 kg/m^2^ in 2018, increasing to 34 m^2^ in 2019, and 33.64 and 34.35 kg/m^2^ in 2020 and 2021 respectively.

The median slaughter age for Ross was 33 days and 46 days for Hubbard. The slaughter age was, like stocking density, not completely stable for Hubbard; in 2017, the median slaughter age for Hubbard was 44 days, while it was 46 days during the rest of the study period.

For all slaughtered broilers, regardless of hybrid, the most common cause of condemnation was ascites, recorded in 222,402 (0.35%) of the 63,209,415 slaughtered carcasses. The second most common cause was discolouration, recorded in 138,428 (0.22%) carcasses, followed by hepatitis in 106,368 (0.17%) carcasses. Skin lesions was recorded as the condemnation cause in 116,814 (0.19%) carcasses while 175,082 (0.28%) carcasses that were small were condemned and 13,052 (0.02%) carcasses were condemned due to fractures.

**Figure 2:**
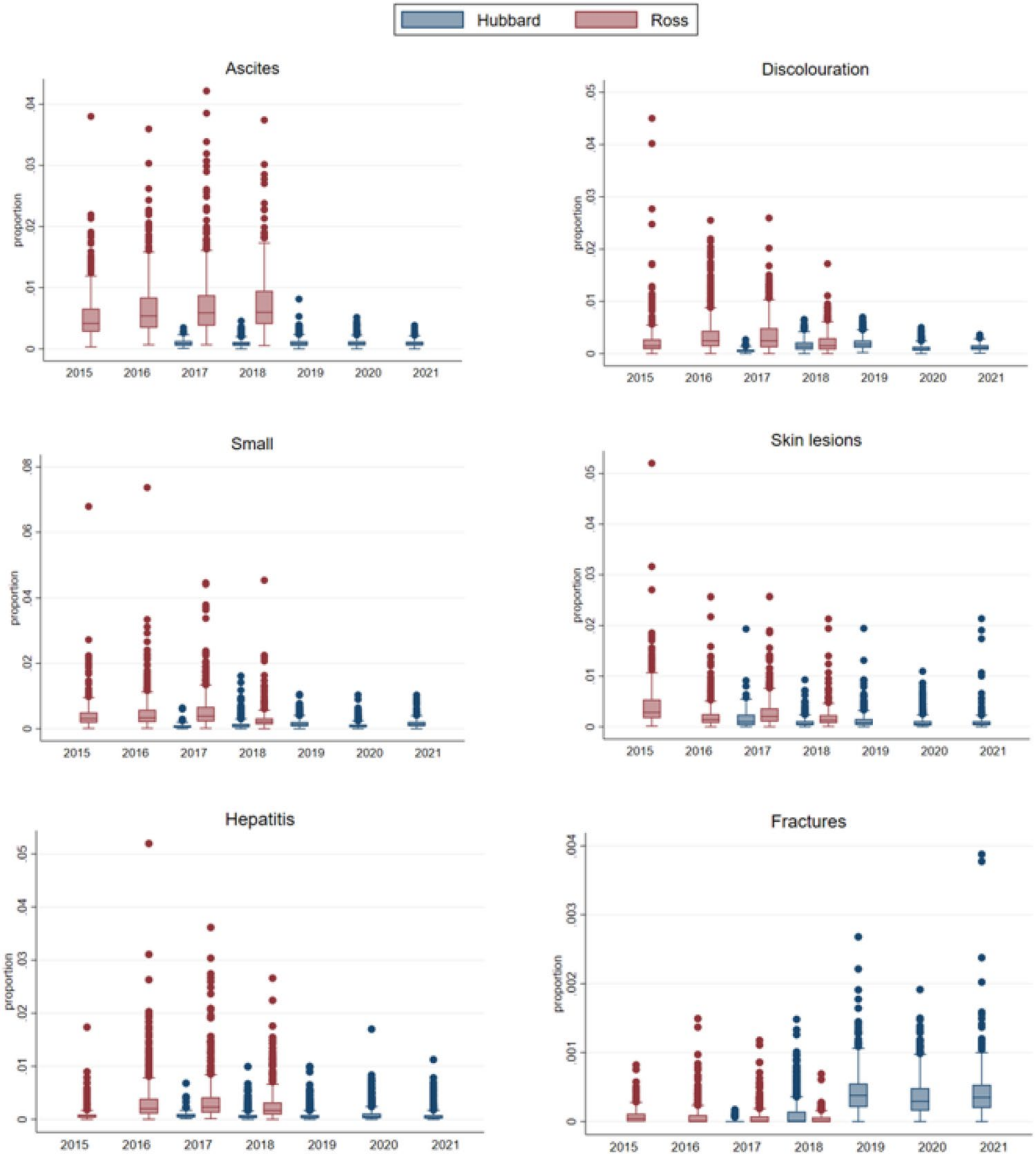
Boxplots showing within-slaughter batch prevalence for each of the six condemnation causes (selected for further analysis) per year, comparing the frequency distributions between the two hybrids Hubbard JA787 (n = 2436 slaughter batches) and Ross 308 (n = 1859 slaughter batches).

The prevalence of condemned carcasses was higher for Ross than for Hubbard, with 623,493/28,804,890 (2.17%) and 221,818/34,404,525 (0.65%) condemnations respectively. The frequency distribution of the within-batch prevalence of the selected condemnation causes throughout the study period is visualised in Fig. 2.

The overall prevalence of the different condemnation causes stratified by hybrid is shown in Table 3. For the fast-growing broiler Ross, ascites was by far the most common condemnation cause recorded in 190,537/28,804,890 (0.66%) of all broilers delivered to slaughter, followed by small in 129,538 (0.45%), discolouration in 89,770 (0.31%), hepatitis in 79,583 (0.28%) and skin lesions in 78,713 (0.27%) of the 28,804,890 broilers. For the slower growing Hubbard, discolouration was the most common condemnation cause recorded in 48,658/34,401,525 (0.14%) of all broilers delivered to slaughter, followed by small in 45,544 (0.13%), skin lesions in 38,101 (0.11%), ascites in 31,865 (0.09%) and hepatitis in 26,785 (0.08%) of the 34,401,525 broilers. For the condemnation causes ascites, discolouration, hepatitis, small and skin lesions, the median within-batch prevalence and the overall prevalence were both higher for Ross compared to Hubbard, shown in Tables 3 and 4, respectively. Fractures was the cause of condemnation in 11,170/34,401,525 (0.03%) Hubbard broilers and 1882/28,804,890 (0.01%) Ross broilers. Median within-batch prevalence of fractures was 0.32 per thousand birds for Hubbard, and 0.07 per thousand birds for Ross and was thus the only condemnation cause more prevalent in Hubbard.

**Table 3:** The overall, and relative frequency, of different causes of condemnations, registered at the abattoir for Hubbard JA787 and Ross 308, between January 2015 and June 2021 (n = 63,209,415 slaughtered broilers, n = 139 farms).

Condemnation cause	Hubbard			Ross	
	n = 34,404,525			n = 28,804,890	
	Number of broilers	Percent		Number of broilers	Percent
Ascites	31,865	0.093		190,537	0.661
Small	45,544	0.132		129,538	0.450
Discolouration	48,658	0.141		89,770	0.312
Skin lesions	38,101	0.111		78,713	0.273
Hepatitis	26,785	0.078		79,583	0.276
Fractures	11,170	0.032		1882	0.007
Peritonitis	15,283	0.044		45,234	0.157
Arthritis	2714	0.008		4694	0.016
Lesions/bleeding	583	0.002		2106	0.007
Tumours	1096	0.003		367	0.001
Cardiac disease	5	< 0.001		29	< 0.001
Other rejects	14	< 0.001		1040	0.004
Total rejects	221818	0.645		623493	2.165

**Table 4:** Relative frequency of condemnation causes within-slaughter batch recorded at the abattoir for Hubbard JA787 and Ross 308, between January 2015 and June 2021 (n = 2436 and 1859 slaughter batches respectively).

Condemnation cause	Hubbard	IQR		Ross	IQR	
	Median (%)	Q1 (%)	Q3 (%)	Median (%)	Q1 (%)	Q3 (%)
Ascites	0.08	0.05	0.12	0.54	0.34	0.85
Fractures	0.03	0.01	0.05	< 0.00	0.00	0.01
Peritonitis	0.03	0.01	0.06	0.12	0.07	0.21
Cardiac disease	< 0.00	0.00	0.00	< 0.00	0.00	0.00
Skin lesions	0.06	0.03	0.13	0.17	0.09	0.32
Arthritis	0.01	0.00	0.01	0.01	0.00	0.02
Lesions/bleeding	< 0.00	0.00	0.00	< 0.00	0.00	0.01
Hepatitis	0.04	0.02	0.09	0.16	0.08	0.33
Discolouration	0.12	0.07	0.19	0.21	0.11	0.40
Small	0.09	0.05	0.16	0.32	0.18	0.54
Tumours	< 0.00	0.00	0.01	< 0.00	0.00	0.00
Other rejects	< 0.00	0.00	0.00	< 0.00	0.00	0.00
Total rejects	0.56	0.42	0.77	1.96	1.48	2.67

### Statistical analysis

The results from the negative binomial regression models assessing the effect of hybrid on condemnation causes are shown in Table 5. Results from the models assessing the effect of slaughter age on condemnation causes for each hybrid are shown in Table 6.

**Table 5:** Results of the mixed effects negative binomial models for ascites, discolouration, hepatitis, small, skin lesions and fractures.

Model	Variables	PR	SE	*p*-value
Ascites	Hybridxseason			
	Hubbard winter	Baseline		
	Hubbard spring	0.866	0.028	< 0.001
	Hubbard summer	0.714	0.024	< 0.001
	Hubbard autumn	0.849	0.028	< 0.001
	Ross winter	6.541	0.216	< 0.001
	Ross spring	1.007	0.046	0.880
	Ross summer	1.124	0.053	0.013
	Ross autumn	1.063	0.051	0.201
Discolouration	Hybridxseason			
	Hubbard winter	Baseline		
	Hubbard spring	1.010	0.102	0.824
	Hubbard summer	1.034	0.047	0.468
	Hubbard autumn	1.028	0.046	0.534
	Ross winter	2.138	0.102	< 0.001
	Ross spring	1.036	0.068	0.584
	Ross summer	0.826	0.056	0.005
	Ross autumn	0.805	0.055	0.002
Hepatitis	Hybrid	3.523	0.113	< 0.001
Small	Hybrid	3.294	0.085	< 0.001
Skin lesions	Hybridxdensity			
	Hubbard density 0	Baseline		
	Hubbard density 1	1.145	0.060	0.009
	Hubbard density 2	1.216	0.064	< 0.001
	Ross density 0	2.901	0.156	< 0.001
	Ross density 1	1.038	0.080	0.631
	Ross density 2	1.143	0.086	0.074

All models included random effects for farm and flock. In the ascites and discolouration models a hybrid—season interaction term was included (winter: December, January and February, spring: March, April and May, summer: June, July and August, autumn: September, October, and November). In the skin lesions and fractures models, interaction between hybrid and stocking density was included (density 0 = lowest and density 2 = highest). The study sample consisted of 139 farms, 4271 flocks and 4295 batches slaughtered from January 2015 to June 2021. Due to missing observations, models including density were fit to 3861 batches from 139 farms.

**Table 6:** Results table showing the estimated effect of slaughter age on the condemnation causes: ascites, discolouration, hepatitis, small, skin lesions and fractures.

Hubbard					Ross				
Variables	n = 2 436 (days)	PR	SE	*p*-value	Variables	n = 1 859 (days)	PR	SE	*p*-value
Ascites	39–45	Baseline			Ascites	28–31	Baseline		
	46	1.002	0.028	0.953		32–33	1.342	0.054	< 0.001
	47–50	1.086	0.031	0.004		34–37	1.835	0.077	< 0.001
Discolouration	39–45	Baseline			Discolouration	28–31	Baseline		
	46	1.143	0.042	< 0.001		32–33	1.558	0.101	< 0.001
	47–50	1.365	0.050	< 0.001		34–37	2.726	0.183	< 0.001
Hepatitis	39–45	Baseline			Hepatitis	28–31	Baseline		
	46	1.040	0.054	0.454		32–33	2.383	0.179	< 0.001
	47–50	1.054	0.056	0.320		34–37	3.322	0.257	< 0.001
Small	39–45	Baseline			Small	28–31	Baseline		
	46	0.931	0.041	0.108		32–33	0.895	0.054	0.065
	47–50	0.944	0.043	0.200		34–37	0.720	0.045	< 0.001
Skin lesions	39–45	Baseline			Skin lesions	28–31	Baseline		
	46	0.946	0.049	0.281		32–33	0.684	0.048	< 0.001
	47–50	0.920	0.049	0.117		34–37	0.681	0.050	< 0.001

Mixed effects negative binomial models were built for the two hybrids separately using each of the condemnation causes as outcomes.

### The effect of hybrid on causes of condemnation

When comparing models, the largest estimated effect of hybrid was found for ascites, where the estimated prevalence was higher in the fast-growing Ross than in the slower growing Hubbard. There was a significant interaction term between season and hybrid, i.e., the effect of hybrid was not the same across all seasons. The largest estimated difference was during winter, with a prevalence 6.5 times higher for Ross than for Hubbard (PR: 6.54, 95% CI 6.13–6.98) (Table 5). The estimated seasonal effect was stronger for Ross than for Hubbard. The largest proportion of unexplained variance in ascites was found between slaughter batches (VPC mean: 44.9% (Hubbard) and 35.1% (Ross)), followed by flock (VPC mean: 31.8% (Hubbard and (37.4% (Ross)) and farm (VPC mean: 23.3% (Hubbard) and 27.4% (Ross)). As for ascites, the discolouration model also included an interaction term between hybrid and season. The largest estimated difference in prevalence of discolouration was found during spring, where the estimated prevalence was approximately twice as high for Ross than in Hubbard PR (95% CI) 2.24 (2.03–2.46). The largest proportion of unexplained variance in discolouration was found between slaughter batches (VPC mean: 49.5% (Hubbard) and 47.6% (Ross)), followed by flock (VPC mean: 31.8% (Hubbard) and 37.4% (Ross)) and farm (VPC mean: 2.3% (Hubbard) and 2.7% (Ross)).

The model estimating the effect of hybrid on skin lesions included an interaction between hybrid and density, and the estimated prevalence at the lowest stocking density (< 32.26 kg/m^2^) was approximately 3 times higher in Ross compared to Hubbard (PR: 2.90, 95% CI 2.61–3.22). The estimated prevalence of skin lesions increased with increasing stocking density for both hybrids, but more for Ross than for Hubbard. The highest estimated prevalence of skin lesions was found for Ross at stocking densities higher than 34.39 kg/m^2^. The largest proportion of unexplained variance in skin lesions was also found between flocks (VPC mean: 81.6% (Hubbard), 84.9% (Ross) followed by farm (VPC mean: 12.8% (Hubbard), 13.3% (Ross)) and slaughter batch (VPC mean: 5.6% (Hubbard), 1.8% (Ross)).

The models estimating the effect of hybrid on hepatitis and small, showed higher estimated prevalence for Ross compared to Hubbard, PR (95% CI) 3.52 (3.30–3.75) and 3.29 (3.13–3.47) respectively. In hepatitis, the largest proportion of unexplained variance was found between flocks (VPC mean: 52.4% (Hubbard), 54.7% (Ross)), followed by slaughter batch (VPC mean: 30.5% (Hubbard), 27.3% (Ross)) and farm (VPC mean: 17.2% (Hubbard), 18% (Ross)). In small, the largest proportion of unexplained variance was also found between flocks (VPC mean: 55.5% (Hubbard), 57.9% (Ross)), followed by slaughter batch (VPC mean: 28.3 (Hubbard), 25.1% (Ross)) and farm (VPC mean: 16.3% (Hubbard), 17% (Ross)).

### The effect of age on causes of condemnation

Table 6 shows the estimated effect of slaughter age on the condemnation causes: ascites, discolouration, hepatitis, small and skin lesions for each hybrid. For Ross the age at slaughter was associated with the condemnation causes ascites, discolouration, and hepatitis when 28–31 days was used as baseline: For ascites the estimated prevalence increased with increasing age, PR (95% CI) 1.34 (1.23–1.45) and 1.84 (1.68–1.99) for the age categories 32–33 days and 34–37 days, respectively. For discolouration, we estimated increasing prevalence with increasing age at slaughter, PR (95% CI) 1.56 (1.37–1.77) and 2.73 (2.39–3.11) for age categories 32–33 days and 34–37 days, respectively. Age at slaughter also had an effect on hepatitis in Ross, where the estimated prevalence was highest in the middle age category (32–33 days) PR (95%CI): 3.32 (2.85–3.87) compared to the lowest age category (28–31 days). The only outcome that showed a decrease in prevalence with increasing age was small, were the PR (95% CI) was 0.90 (0.79–1.01) and 0.72 (0.63–0.81) respectively for age categories 32–33 days and 34–37 days, respectively, compared to the youngest age group (28–31 days).

Although less notable, there was also an effect of increasing age at slaughter for Hubbard for discolouration, PR (95% CI) 1.14 (1.06–1.23) and 1.37 (1.27–1.47) for age categories 46 days and 47–50 days, respectively, compared to the baseline of 39–45 days. For Hubbard, age was not shown to have a significant effect on the estimated prevalence of ascites, hepatitis, small and skin lesions. As age at slaughter does not overlap between hybrids the age effects are not directly comparable between hybrids.

### Sensitivity analysis

For all the investigated condemnation codes, the estimated prevalence ratios were higher in the transition period 2017–2018. The estimated prevalence ratio for ascites in the transition period was more than 30% higher than for the entire study period, PR (95% CI) 8.81 (7.79–9.99). For discolouration, hepatitis and small the estimated prevalence ratio was 15–20% higher in 2017–2018, PR (95% CI) 2.48 (2.09–2.95), 4.13 (3.74–4.56) and 3.98 (3.59–4.41) respectively. For skin lesions, the change in the coefficients was negligible (< 5%), data not shown.

## Discussion

This study aimed to describe causes of carcass condemnations in the broiler hybrids Ross 308 and Hubbard JA787, and to estimate the effect of hybrid on the most common condemnation causes. Furthermore, the effect of hybrid was adjusted for the confounders season and density, and effect of slaughter age was explored for each hybrid separately.

The five most common causes (ascites, discolouration, hepatitis, small, skin lesions) were all more prevalent in the fast-growing Ross (Table 5). Fractures was the only cause of condemnation more prevalent in Hubbard compared to Ross.

Ascites represented more than 30% of the condemnations in Ross and almost 15% of the condemnations in Hubbard. Ascites is the end stage of elevated pressure in the pulmonary circulation and is characterized by accumulation of serous fluid in the coelomic cavity. Imbalance between oxygen supply and the oxygen required to sustain rapid growth rates and high feed efficiencies is believed to be the primary cause of ascites. This imbalance can lead to right ventricular failure with increased venous pressure causing high protein lymph to escape more easily into the abdominal cavity^21^. Ascites develops gradually and might compromise the bird’s welfare over a long time period. The condemnation prevalence of ascites in Ross is in line with the findings in recent studies in Finland and Germany^22,23^. Very few studies have reported the frequency of ascites in slower growing broilers. The association between ascites and rapid growth has; however, previously been described^24–26^, and Baghbanzadeh and Decuypere^25^ state that selective breeding for rapid growth and low feed conversion ratio puts a high demand on metabolic processes and oxygen supply. In 2021, Baxter et al. compared condemnations in the slower growing Hubbard Redbro and Ross 308, and reported higher frequency of ascites in Ross 308^15^. In the present study we report a seasonal effect, with the estimated prevalence of ascites being highest during winter and lower in the summer for both hybrids; however, with a more pronounced seasonal effect in Ross compared to Hubbard. The finding of seasonal variations in the prevalence of ascites is not surprising, as earlier studies have shown that higher oxygen demand in a cold climate might increase the risk of ascites^23,25^. The smaller seasonal effect on Hubbard suggests that slower growing hybrids are less sensitive to seasonal changes than faster growing broilers. The findings of the present study support high growth rate as a major contributor to development of ascites.

The estimated prevalence of condemnations due to growth retardation, resulting in broilers being considerably smaller than their conspecifics were more than three times higher in Ross than in Hubbard (Table 5). This was somewhat surprising, as previous research has found higher frequencies of runts and lower uniformity in slower growing broilers^15,27^, which might be an effect of gender difference causing reduced uniformity with increasing age in mixed-sex flocks^28^. An explanation for our findings might be different causes of growth retardation in broilers differing in growth rates. One factor could be that slower growing broilers are less hungry and spend less time eating^29^, leaving more time and space at the feeders for broilers that for some reason are smaller or “slower” than the rest, to get sufficient nutrition and even up. The slower growing broilers were kept longer and grew larger (Table 2) and because the maximum stocking density is measured in kg/m^2^ at day of slaughter, the Hubbard flocks have fewer individuals. This in turn, will lead to increased availability at the feeders and drinkers. Another likely explanation is the lower disease prevalence in Hubbard, indicated by the lower condemnation numbers, and hence, fewer individuals with growth retardation caused by underlying disease. Vasdal et al.^30^ found poorer flock uniformity with increasing mortality, suggesting that uniformity is associated with broiler health status.

In the present study, condemnations due to discolouration were more common in Ross compared to Hubbard, although it was the most common cause in Hubbard, found in 21.9% of all condemned carcasses. This condemnation category included deviant colour of the carcass, either darker or lighter colour and a deviant smell of the carcass without detection of any of the other condemnation codes. The main reason for discolouration is quality defects related to myodegenerative disorders, e.g., wooden breast (WB), a condition characterised by accumulation of interstitial connective tissue during regeneration process^3^. There are several indications for a connection between WB and fast growth rate, like greater myofiber diameter, followed by poorer circulation due to reduced space available for capillaries and blood supply in fast-growing broilers^3,18^, and our findings of reduced prevalence in the slower growing broiler are in line with previous research^5,18^. As the meat is condemned, the condition has a considerable economic impact. Furthermore, reports from studies that have scored WB and recorded welfare indicators suggest that myopathies like WB may affect animal welfare^31,32^. Norring et al.^31^ found that broilers affected by WB had higher gait score^31^ and Kawasaki et al.^32^ found that WB inhibits the extensibility of the muscle and therefore limits the broilers’ ability to lift their wings^32^, and Papah et al.^33^ found that affected birds were unable to right themselves from accidental dorsal recumbency^33^. In the present study WB was found more frequently in Ross than in Hubbard, although Hubbard was the heaviest broiler. This may suggest that the causal mechanisms of WB is linked to rapid growth rather than body weight at slaughter.

Hepatitis caused about 12% of the condemnations in both hybrids. However, the proportion of carcasses condemned due to hepatitis was considerably lower for Hubbard than for Ross. Hepatitis is commonly related to infections by *Clostridium perfringens* which causes necrotic enteritis^34^, *Escherichia coli*^35^ and infectious bursal disease virus^36^, and is therefore not directly connected to growth rate. In 2003, Cheema et al.^6^ concluded that genetic selection for improved broiler performance has had a negative impact on the broiler’s antibody response thereby increasing the risk of infectious diseases^6^. Based on this it could be speculated that the present findings might be related to differences in immune response resulting in improved gut health; however, further studies are required to investigate this.

The estimated prevalence of condemnations due to skin lesions was three times higher in the fast-growing compared to the slower growing broilers. This was surprising, as more scratches could be expected in the slower growing broiler due to a higher activity level^16^. A plausible explanation is the protective effect of a better developed plumage in slower growing broilers^5,15^, as good feathering is found to protect against skin scratches^37^. Another reason for the lower prevalence of skin lesions might be that these birds move away from others more easily, thereby avoid being scratched. We found a positive association between skin lesions and stocking density. This is in line with earlier studies, and might be caused by broilers climbing on top of each other because of lack of space, and reduced access to feeders and drinkers^38^. Further investigation is needed to affirm the causal pathways between hybrid and skin lesions. Condemnations due to skin lesions might also in a few cases comprise dermatitis related to breast burns. Breast burn may be caused by moist litter, which is related to stocking density, feed quality, ventilation, bird weight, leg health and genotype^39^. Rayner et al.^16^ reported dryer litter in productions with slower growing hybrids. We did not have access to data about litter quality in this study.

Condemnation due to fractures comprised only fractures with changes in colour and presence of pus, indicating that they were several days old as these are the changes of relevance for food safety. Although being an uncommon cause of condemnation, constituting 1.5% of all condemnations in total (Table 3), it is noteworthy that fractures was the only cause of condemnation more frequent in Hubbard compared to Ross. Although the number of broilers condemned due to fractures was low, the relative difference between the hybrids was notable. One possible explanation is the higher activity level of slower growing broilers making them more prone to injuries and fractures^15,16^. It may be related to increased weight, and therefore more load involved in any movements. There is limited descriptions of fracture occurrence in existing literature, and although uncommon, it is a health issue worth investigating further.

Stockmanship is regarded as the single most important influence of the welfare of farm animals^40^ and low condemnation frequencies might be achieved also in fast growing broilers with good farm management. As can be seen in Fig. 2, there was a considerable variation in the proportion of condemnation causes between batches, especially for Ross. Several of the conditions discussed have a multifactorial aetiology, and differences in management might be part of the explanation to the variation between batches of the same hybrid. Overall, the largest proportion of the unexplained variance across models was found to be within flock or batch for both hybrids (mean VPC range 75–95%), whereas a smaller proportion of the variance was found at farm level (5–25%), suggesting that factors varying between flocks and batches had more impact on the unexplained variation than farmer management per se.

An inherent weakness of observational studies is the possibility of unmeasured confounding. Limitations to the present study was that both hybrids were not present throughout the study period. Thus, we cannot exclude other temporal changes e.g., continued efforts to improve animal health and welfare, as a potential source of bias. To assess this, we performed a sensitivity analysis using only the two years of overlap between the hybrids. Overall, the results from the sensitivity analysis showed that for the shorter period the differences in condemnation frequencies between hybrids were larger compared to the entire study period. We therefore consider the presented estimated effects as conservative. By choosing a study period beyond the transition period we deemed it easier to assess any long-term trends in the occurrence of the outcome variables. A known change was an increase in stocking density for Hubbard in 2019 compared to the previous two years. This might be part of the explanation behind the lower occurrence in fractures in Hubbard in 2017 to 2018 compared to the rest of the study period, visible in Fig. 2. The external validity was deemed good for the source population as few exclusions were made and almost all farmers consent to participate in the study, thus minimizing the risk of selection bias. The results are likely also generalizable to broilers of the same hybrids kept in similar production systems. Nevertheless, further studies are needed to understand the causal pathways between growth rate and several of the condemnation causes, like discolouration, small and fractures. It should also be noted that the recording of only one condemnation code per bird, implies a risk of underreporting for some of the condemnation codes.

Overall, our findings of fewer condemnations in slower growing broilers are in line with previous research^15,16^. However, there is still limited research on slower growing broilers. There is, to the authors knowledge, only one previous large-scale observational study performed in commercial production assessing differences in causes of condemnation between broilers of different growth rates^15^. The differences in condemnation rates found in the present study along with differences in the associations found between hybrid and season, age and density show that existing knowledge about fast growing broilers cannot automatically be generalized to slower growing hybrids.

In conclusion, we found a higher frequency of condemnations in the fast-growing Ross 308 than in the slower growing Hubbard JA787. The frequency of all condemnation causes was higher in Ross compared to Hubbard, except for fractures. The most common cause of condemnation was ascites. For this condition, the largest estimated difference was found during winter where the prevalence was six times higher in Ross 308 compared to Hubbard JA 787. This study showed notable differences between hybrids in prevalence and causes of condemnation, thus showing that broiler hybrid has important implications for animal health, welfare, economy, and sustainability of the value chain.

### Ethical statement

All methods were conducted in accordance with Norwegian regulations. Informed written consent was obtained by all farmers contributing with data to this study. Being a retrospective observational study, no animal intervention was needed, and so ethical approval was not required for this study.

## Supplementary Information

Supplementary Information.

## Data Availability

The data that support the findings of this study are available from Norsk Kylling AS but restrictions apply to the availability of these data, and so they are not publicly available. Data are however available from the authors upon reasonable request and with permission of Norsk Kylling AS.
