# Supplementary material for: Comparison of carcass condemnation causes in two broiler hybrids differing in growth rates
Source: Sci Rep. 2023 Mar 14;13:4195. doi: 10.1038/s41598-023-31422-0 (PMC10015030; doi:10.1038/s41598-023-31422-0)

Comparison of carcass condemnation causes in two broiler hybrids differing in growth rates

Merete Forseth[a]✉, Randi Oppermann Moe[b], Käthe Kittelsen[c], Eystein Skjerve[b], Ingrid Toftaker[b]

a Norsk Kylling AS, Havneveien 43, 7300 Orkanger, Norway
b Norwegian University of Life Sciences, Universitetstunet 3, 1433 Ås, Norway
c Animalia, Norwegian Meat and Poultry Research Centre, Lørenveien 38, 0513 Oslo, Norway

✉*email: merete@norsk-kylling.no*

Supplementary information

Fig S1. The study area consisting of two counties in mid Norway. The locations of study farms (n=139) are shown as black dots. (January 1, 2015, to June 22, 2021).

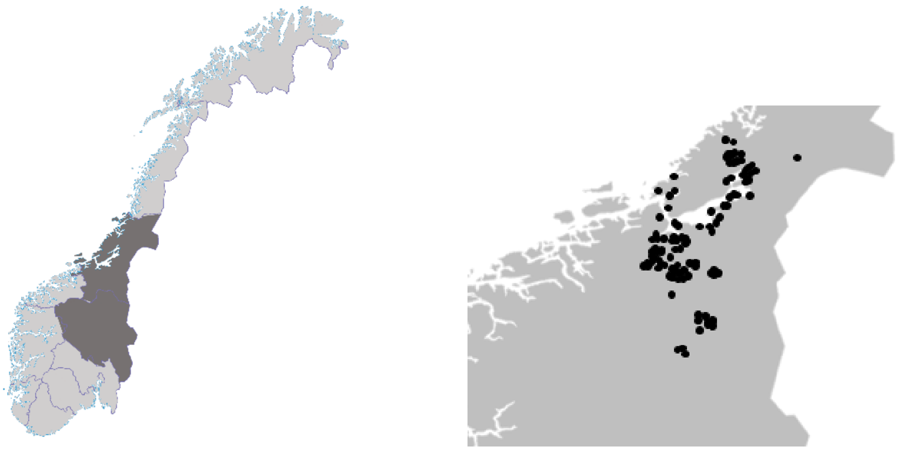

Table S1. The hierarchical structure of the data in a study investigating causes of condemnation in two different hybrids for 4295 batches from 139 Norwegian poultry farms in the time period 2015–2021)

Level	Number	Average number per unit at next-higher level	Range
Farm (highest level)	139	-	
Flock	4271	30.4	1-84
Batch (lowest level)	4295	1.01	1-2

Fig S2. Causal diagram for ascites, discolouration, hepatitis, skin lesions, small and fracture models

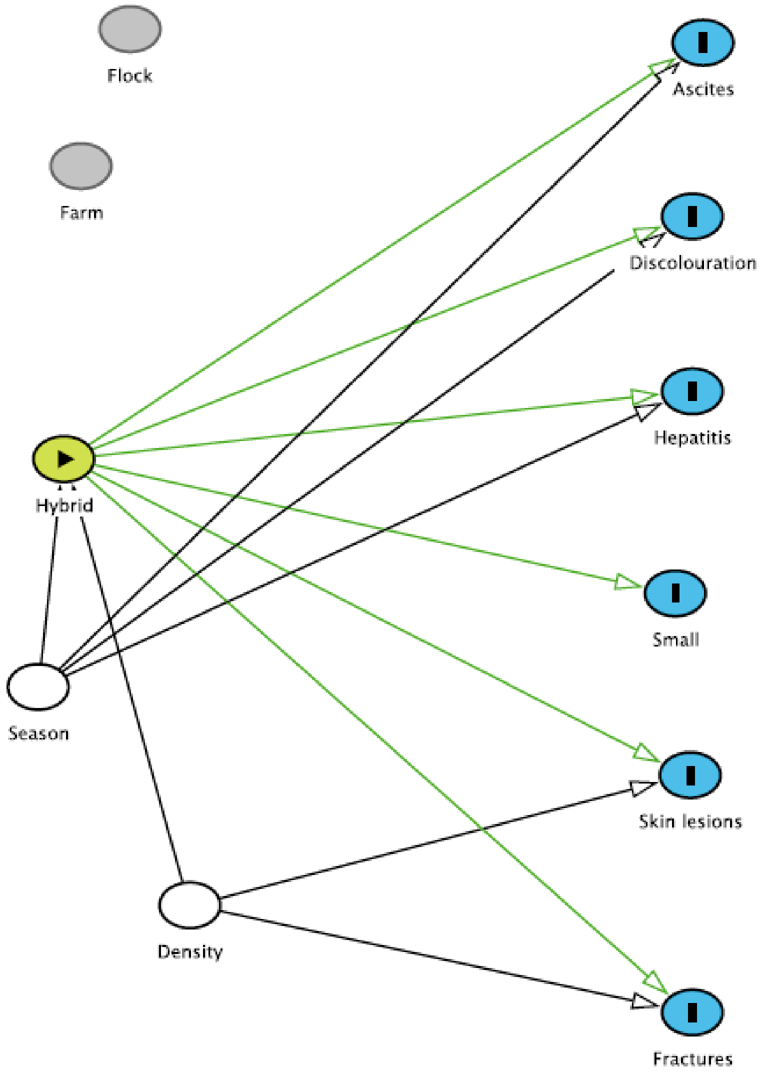

Supplement: Supplementary file 1 — Supplementary Information. [file 41598_2023_31422_MOESM1_ESM.pdf]
